# Surface Roughness and *Streptococcus mutans* Adhesion on Metallic and Ceramic Fixed Prosthodontic Materials after Scaling

**DOI:** 10.3390/ma14041027

**Published:** 2021-02-22

**Authors:** Jenni Hjerppe, Sampo Rodas, Johanna Korvala, Paula Pesonen, Anna Kaisanlahti, Mutlu Özcan, Juho Suojanen, Justus Reunanen

**Affiliations:** 1 Clinic of Reconstructive Dentistry, Center of Dental Medicine, University of Zürich, 8032 Zürich, Switzerland; 2 Biocenter Oulu & Cancer and Translational Medicine Research Unit, University of Oulu, 90014 Oulu, Finland; sampo.rodas@gmail.com (S.R.); johanna.korvala@gmail.com (J.K.); anna.kaisanlahti@oulu.fi (A.K.); justus.reunanen@oulu.fi (J.R.); 3 Infrastructure for Population Studies, Faculty of Medicine, University of Oulu, 90014 Oulu, Finland; paula.pesonen@oulu.fi; 4 Center of Dental Medicine, Division of Dental Biomaterials, Clinic for Reconstructive Dentistry, University of Zürich, 8032 Zürich, Switzerland; mutlu.ozcan@zzm.uzh.ch; 5 Päijät-Häme Joint Authority for Health and Wellbeing, Department of Oral and Maxillo-facial Surgery, 15850 Lahti, Finland; juho.o.suojanen@hus.fi; 6 Cleft Palate and Craniofacial Centre, Department of Plastic Surgery, Helsinki University Hospital, 00029 Helsinki, Finland

**Keywords:** surface roughness, bacterial adhesion, biofilm, *Streptococcus mutans*, zirconia, lithium disilicate, cobalt-chromium, titanium, polishing, curette scaling, ultrasonic scaling

## Abstract

The aim of this study was to evaluate the surface roughness of fixed prosthodontic materials after polishing or roughening with a stainless steel curette or ultrasonic scaler and to examine the effect of these on *Streptococcus mutans* adhesion and biofilm accumulation. Thirty specimens (10 × 10 × 3 mm^3^) of zirconia (Zr), pressed lithium disilicate (LDS-Press), milled lithium disilicate glazed (LDS-Glaze), titanium grade V (Ti) and cobalt-chromium (CoCr) were divided into three groups (n = 10) according to surface treatment: polished (C), roughened with stainless steel curette (SC), roughened with ultrasonic scaler (US). Surface roughness values (Sa, Sq) were measured with a spinning disc confocal microscope, and contact angles and surface free energy (SFE) were measured with a contact angle meter. The specimens were covered with sterilized human saliva and immersed into *Streptococcus mutans* suspensions for bacterial adhesion. The biofilm was allowed to form for 24 h. Sa values were in the range of 0.008–0.139 µm depending on the material and surface treatment. Curette and ultrasonic scaling increased the surface roughness in LDS-Glaze (*p* < 0.05), Ti (*p* < 0.01) and CoCr (*p* < 0.001), however, surface roughness did not affect bacterial adhesion. Zr C and US had a higher bacterial adhesion percentage compared to LDS-Glaze C and US (*p* = 0.03). There were no differences between study materials in terms of biofilm accumulation.

## 1. Introduction

Fixed prosthodontic reconstructions, i.e., inlays, onlays, veneers, crowns and fixed dental prosthesis (FDPs), are used to restore the dentition damaged by tooth wear or dental caries. Ceramic materials like lithium disilicate glass ceramics and zirconia have excellent esthetic and mechanical properties [1,2,3] and can therefore be considered as the material of choice in many clinical cases. However, a metal framework can give durability to the FDP reconstructions, and for esthetic purposes, the framework is fully or partially veneered. Typical metals used for tooth-and implant-borne reconstructions are gold alloys, titanium and cobalt-chromium.

Bacterial plaque is involved in the development of periodontal and peri-implant diseases [4,5] as well as in dental caries [6]. Streptococci species are known to be early colonizers in supragingival plaque [7,8,9]. Streptococci are capable of binding to several host molecules and to other bacterial species and can therefore function as a solid base for biofilm development and maturation [8]. In intraoral conditions all oral surfaces are covered with saliva. The early colonizers in dental plaque attach to the salivary glycoproteins, replicate themselves and grow into microcolonies [8,10]. The bacteria secrete extracellular polymeric substance (EPS), which enables the adhesion between the surfaces and bacteria. When the bacterial cells replicate themselves, the three-dimensional biofilm is formed [10].

Restorative materials can act as adherent sites for oral bacteria. Biological, chemical and physical interactions enable the attachment of oral bacteria to material surfaces [10,11]. Certain material surface properties are considered as important factors in bacterial biofilm development: i.e., surface roughness and topography on micro- and nano-level, surface chemical composition, surface chemical charge, hydrophobicity or hydrophilicity of the surface and stiffness and elasticity of the material [10,12,13]. In vivo studies show differences in bacterial adhesion between different implant abutment materials, zirconia having lower bacteria incidence than titanium [14,15]. Microscale surface roughness caused by the fabrication method or scaling and polishing seem to have a role in bacterial adhesion on lithium disilicate, zirconia and type III gold alloy surfaces in the in vitro study set-ups where saliva was not involved [16,17,18]. When saliva is involved, surface roughness does not seem to have as big of a role in biofilm formation as the material itself [12].

In intraoral conditions prosthodontic materials can be exposed to mechanical wear during the maintenance care of the dentition. When cementing tooth-borne fixed prostheses or single crowns, the cement residuals are typically removed with a stainless steel curette. Additionally, periodontal diseases are common [19,20,21], and in periodontal maintenance care, ultrasonic scalers with a stainless steel tip and stainless steel curettes are used for removing calculus and bacterial plaque [22]. These instruments could also be used on supra-mucosal implant-borne structures.

There is a lack of information on scaling-induced surface roughness and bacterial adhesion when human saliva is present. Therefore, the aim of this study was to evaluate surface roughness of certain commonly used fixed prosthodontic materials after polishing or roughening with a stainless steel curette or ultrasonic scaler in order to mimic the removal of dental calculus or cement. The second aim was to evaluate the bacterial adhesion and biofilm accumulation of *Streptococcus mutans* on these materials with different surface treatments and exposed to human saliva. The null hypothesis was that there is no difference in surface roughness between different materials after the surface treatments. The second null hypothesis was that there is no difference in bacterial adhesion or biofilm formation between the different materials with different surface treatments.

## 2. Materials and Methods

### 2.1. Specimen Preparation

Thirty square shaped specimens (10 × 10 × 3 mm^3^) of partially yttrium stabilized zirconium dioxide, zirconia (Zr) (Kyocera StarCeram Z-Al-Med, Selb, Germany), pressed lithiumdisilicate (LDS-Press) (e.max Press A2, Ivoclar Vivadent, Schaan, Lichtenstein), milled lithiumdislicate glazed (LDS-Glaze) (e.max CAD A3, Ivoclar Vivadent, Schaan, Lichtenstein), polished titanium grade V (Ti) (Ti6Al4V ELI-ASTM F136, PSM Medical solutions, Gunningen, Germany) and polished cobalt chromium (CoCr) (Zenotec NP, Wieland Dental + Technik GmbH & Co. KG, Pforzheim, Germany) were prepared by accomplished dental technicians. The study groups and detailed manufacturing procedures are presented in Table 1. All the specimens were highly polished on one side according to the material specific scheme (Table 2). After the polishing procedures, the specimens were steam cleaned. Additionally, specimens in the LDS-Glaze group were glazed with IPS e.max CAD Crystall Glaze spray (Ivoclar, Vivadent, Schaan, Lichtenstein). Two layers of spray was applied on the polished specimen surface and the specimens were crystallized in a specific oven (Programat P300, Ivoclar Vivadent, Schaan, Lichtenstein) according to manufacturer’s instructions.

All the material groups were divided in three subgroups (*n* = 10) according to the surface treatments mimicking the removal of dental calculus/cement: control (polished surface) (C), roughened with stainless steel curette (SC), roughened with ultrasonic scaler (US). One author (J.H.) completed all the surface treatments of the specimens. Subsequently, the specimens in group C were left as such with polished surfaces. In group SC, 10 specimens/material were placed on a silicone mold (Coltoflax Putty, Coltene Whaledent, Altstätten, Switzerland) and the previously polished surface was manually roughened with a stainless steel curette (Gracey P3–P4, LM Instruments, Parainen, Finland) using a force corresponding to 5 N. In group US, 10 specimens/material were placed on a silicone mold (Coltoflax Putty, Coltene Whaledent, Altstätten, Switzerland) and the previously polished surface was manually roughened with an ultrasonic scaler (EMS Piezon 250, E.M.S. Electro Medical Systems S.A., Nyon, Switzerland) using a low setting (3/8) with water cooling and a force corresponding to 1N. The specimens were roughened systematically throughout the whole polished surface by making five even strokes at one end of the surface and then making five strokes next to the first ones. This procedure was repeated until the whole surface was roughened. The calibration of the operator for corresponding forces was done with the help of a Correx Tension Gauge (Haag-Streit Diagnostics, Köniz, Switzerland). The operating part of the instrument was used at a 15-degree angle to the specimen. For SC groups, the instrument was changed after five specimens, and for US groups, the tip of the ultrasound scaler was changed after every group.

After the surface treatments with scalers or an ultrasonic device, all the specimens were ultrasonically cleaned in distilled water for 15 min.

### 2.2. Surface Roughness Measurement

The surface roughness of the specimens was measured with a spinning disc confocal microscope with a white light source (COM, μSurf explorer, NanoFocus, Oberhausen, Germany). The parameters Sa (arithmetical mean height) and Sq (root mean square height) were determined with a 100× magnification lens, and a cutoff wavelength of 80 μm was used. The vertical resolution of the lens was 2 nm, and for the measurement area of 160–158 μm, the numerical aperture was 0.8–0.95. At least two readings/specimen were made at randomly selected areas and the mean and median values in micrometers were calculated. From the surfaces treated with a stainless steel curette or ultrasonic scaler, the depth of the randomly selected notches caused by the curetting instrument was also measured if they were detectable.

### 2.3. Contact Angle and Surface Energy Measurements

Four specimens/group were randomly selected and used for measuring the equilibrium contact angles (*θ_c_*) according to the previously introduced sessile drop method [23]. The measurements were completed with a contact angle meter (EasyDrop, Krüss GmbH, Hamburg, Germany). Milli-Q-Water and diiodomethane (Sigma-Aldrich, St.Louis, USA) were used as liquids for contact angle measurements. Surface tension (IFT) values for Milli-Q-water were 72.8 mN/m (Disperse Pt 21.8, Polar Pt 51, Acid Pt 25.5, Base Pt 25.5) and for diiodomethane 50.8 mN/m (Disperse Pt 50.8, Polar Pt 0, Acid Pt 0, Base Pt 0). Three drops/specimen/liquid were measured and the mean value was calculated. Surface energy (SFE) was calculated using the Owens-Wendt approach [24].

### 2.4. Energy-Dispersive Analysis

Energy-dispersive X-ray analysis (EDX) (Oxford Instruments X-Max) was performed using a scanning electron microscope (Zeiss Ultra Plus) to determine the elemental composition of the study materials. One specimen from each surface treatment group of ceramic materials (Zr, LDS-Glaze, LDS-Press) (three specimens/material) was ultrasonically cleaned and covered with carbon (Jeol JEE-420) before analysis. One specimen from each surface treatment group of metals (Ti and CoCr) (three specimens/material) was ultrasonically cleaned and analyzed as such. The EDX analysis was carried out using an acceleration voltage of 15 kV and a magnification of 100×. A 20 s spectra collection time was used to determine the elemental composition of the study materials. Two recordings were made for each specimen. The wt% of elements were analyzed with EDX computer software (Oxford Instruments AZtec).

### 2.5. Saliva Contamination

Paraffin-wax stimulated saliva was collected from four healthy volunteers. The collected saliva was then pooled and filtered through a 0.45 μm filter (#FPE-404-150, Jet Biofil) to produce sterile saliva. The saliva was stored in aliquots at −80 °C. Before usage, the saliva was diluted 1:1 with 1× phosphate buffered saline (PBS).

Prior to the bacterial adhesion tests, the specimens were autoclaved and then exposed to saliva-PBS mixture for 30 min at room temperature in 24-well cell culture plates (#3524, Corning Incorporated). The saliva suspension was then removed using suction and the specimens were immersed in 1× PBS.

### 2.6. Cell Cultivation and Adhesion

*Streptococcus mutans* (SM) DSM 20,523 was bought from Leibniz Institute DSMZ–German Collection of Micro-organisms. The cells were cultured in DSMZ 92 medium (trypticase soy broth supplemented with 0.3% yeast extract) and grown aerobically without agitation overnight at +37 °C. For the adhesion tests, the cells were diluted to 1:50 with DSMZ 92 medium and metabolically labelled by adding 8.4 μL/mL [5′-^3^H] thymidine (20.0 Ci/mmol, Perkin Elmer, Boston, MA, USA) to the cell suspension. The cells were grown again overnight at +37 °C. The cell suspension was centrifuged at 16,000 g for 5 min at + 21 °C and washed once with 1xPBS and then diluted to OD_600_ = 0.25 with 1× PBS. Next, 600 μL of cell suspension was placed on each specimen on a 24-well plate. The plates were then sealed with parafilm and incubated at +37 °C for 1 h. Nine specimens/group were used for cell adhesion tests.

The specimens were moved to a new sterile 24-well plate and washed 3 times with sterile 1× PBS to remove any excess thymidine and non-adhered bacteria. The adhered cells were collected by scraping the specimens with a dental micro-brush (Quick-Stick #87110, Dentonova AB). Each specimen was scraped with three different brushes using a zigzag-like maneuver from top to bottom. After each brush the specimens were turned 90° to ensure full scraping coverage of the surface. The brush heads of each stick were cut into a 20 mL plastic vial (#216-4306 HDPE, VWR Int., Radnor, PA, USA) filled with 3 mL of liquid scintillation agent (Optiphase Hisafe 3, Perkin Elmer, Waltham, MA, USA) followed by vortexing and scintillation counting (Tri-Carb 2900TR, Perkin Elmer, Shelton, USA). Controls were prepared using 100 μL of the previously prepared OD_600_ = 0.25 cell suspension.

### 2.7. Biofilm Formation

For the biofilm formation experiments, the bacterial cells were grown and the saliva contamination was performed as described above with the adhesion tests, except without adding thymidine before the second o/n incubation. Biofilm accumulation was done by incubating the cells in DSMZ 92 medium on the specimens for 24 h at +37 °C. Non-adherent bacteria were removed by washing the specimens three times with 1xPBS. The biofilm was collected with three micro-brushes (#18-904B Premium Plus, Bournemouth, UK) as described earlier, and the tips were cut into an Eppendorf tube with 1 mL of 1xPBS followed by vortexing to detach the biofilm from the brushes. To enumerate the adhered bacteria in colony forming units (CFU), serial 1:10 dilutions were made and plated on Medium 92 agar plates, which were incubated for 48 h at +37 °C prior to CFU counting. Four specimens/group were studied in this test.

### 2.8. Statistical Analysis

Statistical analysis was performed using IBM SPSS Statistics (Version 26). *p*-values of <0.05 were considered statistically significant.

The normality of the target variables was evaluated using the box-plots, Shapiro-Wilk and Kolmogorov-Smirnov tests. Descriptive statistics for surface roughness, Sa and Sq, and the mean depth of the scratches on the surface are presented as medians and interquartile ranges. Differences in surface roughness, scratch depth, adhesion percentage and CFU values between three surface treatment groups (control, SC and US) by material were first tested with a Kruskall-Wallis test. To compare differences between two groups, the comparisons were continued with a Mann-Whiney U test. Bonferroni correction was used for the results between two groups.

The bivariate correlations of surface roughness Sa and Sq with the bacterial adhesion percentage (adhesion %) and CFU between treatment groups by material were determined using Spearman’s correlation coefficients. The adhesion percentage was calculated using the specimens’ and controls’ scintillation counts.

Mean values and standard deviations of contact angles were calculated. Contact angle data was analyzed using a Chi-Square test, and the differences between different materials were compared. Correlation coefficients were calculated to evaluate associations between the rate of bacterial adhesion and contact angles.

Mean values and standard deviations of surface energy were calculated, and these values were compared between the surface treatment groups by material using a calculation of analysis of variance from summary data including Tukey’s post-hoc test (https://statpages.info/anova1sm.html, accessed on 28 August 2020).

## 3. Results

### 3.1. Surface Roughness Measurements

**Figure 1:**
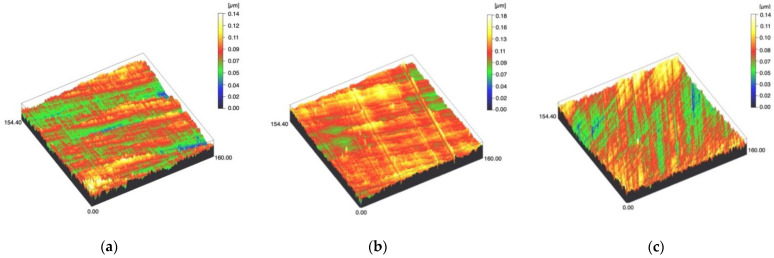
Spinning disc confocal microscope images of the study specimens with vertical resolution of 2 nm: (**a**) Zr C, (**b**) Zr SC, (**c**) Zr US.

**Figure 2:**
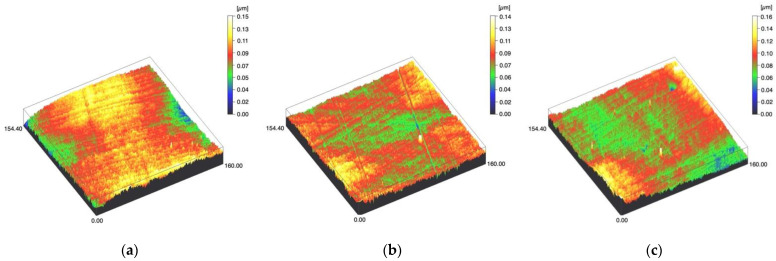
Spinning disc confocal microscope images of the study specimens with vertical resolution of 2nm: (**a**) LDS-Press C, (**b**) LDS-Press SC, (**c**) LDS-Press US.

**Figure 3:**
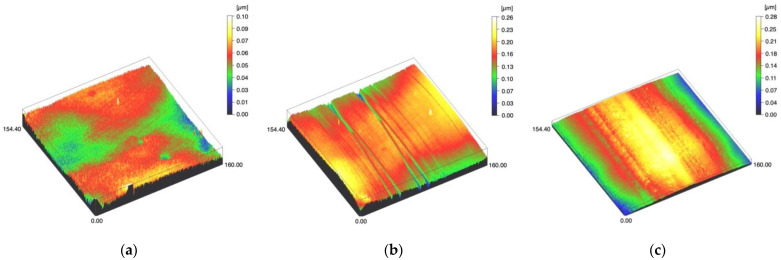
Spinning disc confocal microscope images of the study specimens with vertical resolution of 2nm: (**a**) LDS-Glaze C, (**b**) LDS-Glaze SC, (**c**) LDS-Glaze US.

**Figure 4:**
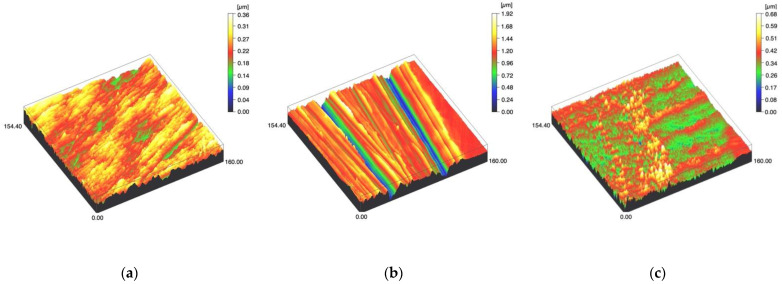
Spinning disc confocal microscope images of the study specimens with vertical resolution of 2nm: (**a**) Ti C, (**b**) Ti SC, (**c**) Ti US.

**Figure 5:**
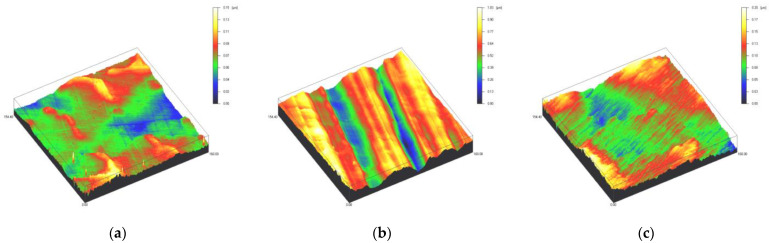
Spinning disc confocal microscope images of the study specimens with vertical resolution of 2nm: (**a**) CoCr C, (**b**) CoCr SC, (**c**) CoCr US.

**Figure 6:**
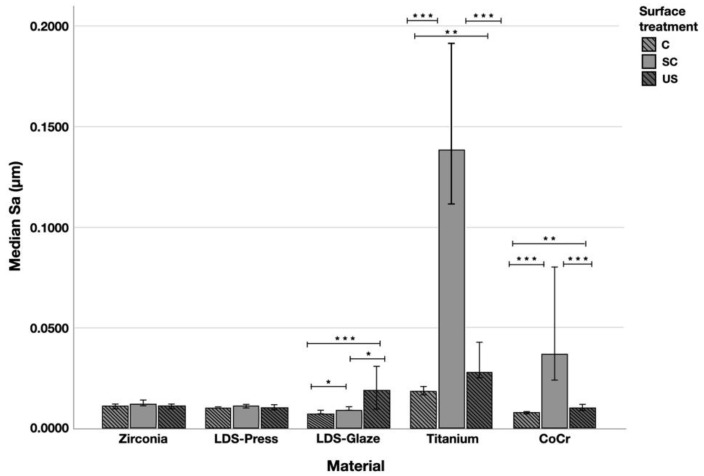
The median surface roughness (SD) values Sa (μm) with interquartile ranges (IQR) of the study specimens without surface treatment (control C), after scaling with a curette (SC) and after ultrasonic scaling (US). Statistical differences within a material are marked with lines * *p* < 0.05, ** *p* < 0.01, *** *p* < 0.001.

Surface profiles of the study specimens are presented in spinning disc confocal microscope images (Figure 1, Figure 2, Figure 3, Figure 4 and Figure 5). The median surface roughness Sa (arithmetical mean height) values varied in the range of 0.008–0.139 µm depending on the material and surface treatment subgroup, and detailed results of the median surface roughness values Sa (μm) with interquartile ranges (IQR) are presented in Figure 6. Significant differences in surface roughness Sa values were found between specimens with different surface treatment subgroups (C, SC and US) in LDS-Glaze (*p* < 0.05), Ti (*p* < 0.01) and CoCr groups (*p* < 0.001). In Ti SC and CoCr SC groups, scaling provided greater roughness values, but in the LDS-Glaze US group, ultrasonic scaling resulted in greater roughness. When comparing the different materials, Ti showed greater Sa values compared to all the other materials in C and SC subgroups (*p* < 0.001). The same observation was made in the US subgroup (*p* < 0.001), except for LDS-Glaze material in which the Sa value did not differ from that of Ti (*p* = 0.84).

**Figure 7:**
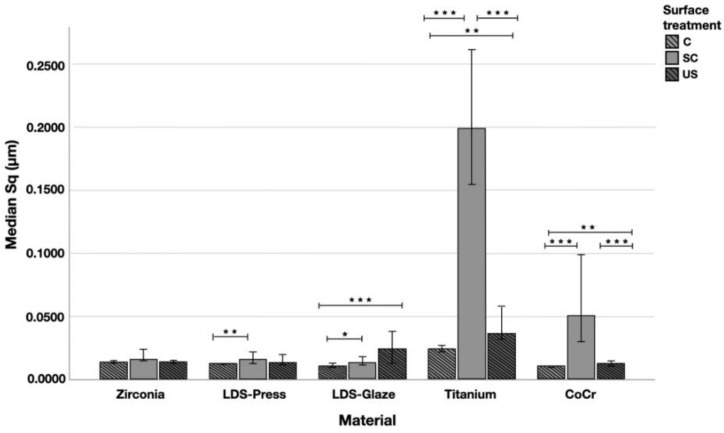
The median surface roughness (SD) values Sq (μm) with interquartile ranges (IQR) of the study specimens without surface treatment (control C), after scaling with a curette (SC) and after ultrasonic scaling (US). Statistical differences within a material are marked with lines * *p* < 0.05, ** *p* < 0.01, *** *p* < 0.001.

The median Sq (root mean square height) values varied in the range of 0.011–0.199 µm depending on the material and surface treatment subgroup (Figure 7). Significant differences between all surface treatment subgroups per material were seen in Ti (*p* < 0.015) and CoCr (*p* < 0.006). LDS-Glaze specimens’ Sq values were significantly higher in SC and US groups compared to control specimens (*p* = 0.03). LDS-Press specimens’ Sq values were significantly higher in the SC group compared to C specimens (*p* = 0.003). For the Zr group no significant difference between the different surface treatments was seen (*p* = 0.144). When comparing the different materials and the surface treatment subgroups, the greatest Sq values were measured from Ti (*p* < 0.001), however, in the US subgroup there was no significant difference between Ti and LDS-Glaze (*p* = 0.201).

The median depth of scratch varied in the range of 0.047–0.778µm. Curette scaling created deeper scratches than ultrasonic scaling or polishing (control) in Zr, LDS-Press, Ti and CoCr specimens (*p* < 0.001). No difference was detected between SC and US scratch depth in LDS-Press, both being significantly greater than in the C group (*p* < 0.001, *p* = 0.018 respectively). In LDS-Glaze there were no significant differences in scratch depth between the surface treatment subgroups (*p* = 0.458). Between the different materials, Ti specimens showed the deepest scratches (*p* < 0.05 in C and US groups, *p* < 0.001 in SC groups).

### 3.2. Energy-Dispersive Analysis

Chemical elements of studied materials according to energy-dispersive X-ray analysis (EDX) are presented in Table 3. The sensitivity of the EDX was not enough for detecting lithium in the groups LDS-Glaze and LDS-Press as the elemental mass of Li was too small.

### 3.3. Contact Angle and Surface Free Energy Measurements

**Figure 8:**
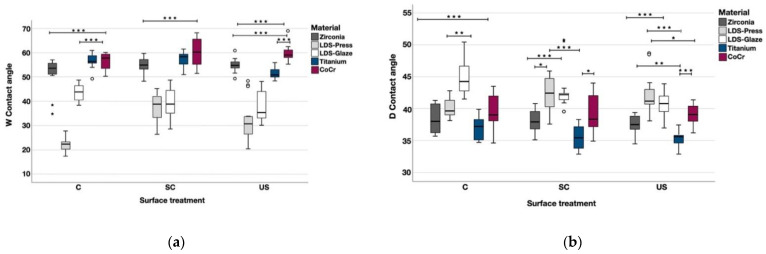
The median contact angle measurements with interquartile ranges (IQR) between the materials within different surface treatments with (**a**) water, (**b**) diiodomethane. Statistical differences between materials within each surface treatment are marked with lines. * *p* < 0.05, ** *p* < 0.01, *** *p* < 0.001.

The contact angle measurements were completed with two different liquids and the values are shown in the Table 4. When tested with water, Zr, LDS-Glaze and CoCr showed no significant difference between different surface treatments. Within each material, LDS-Press C had a lower contact angle compared to SC (*p* < 0.001) and US (*p* = 0.006) subgroups. Ti US had a lower contact angle compared to C (*p* = 0.003) and SC (*p* = 0.003). When tested with diiodomethane Zr, LDS-Press, Ti and CoCr showed no significant difference in contact angles between different surface treatments within each material. LDS-Glaze US had a lower contact angle compared to LDS-Glaze C (*p* = 0.003) and no difference compared to LDS-Glaze SC. The statistical comparison between different materials within each surface treatment is presented in Figure 8a and b. When tested with water, LDS-Press and LDS-Glaze showed the lowest contact angle values (*p* < 0.001), whereas Zr Ti and CoCr showed the highest values. The opposite trend was seen when the contact angles were measured with diiodomethane (Figure 8b).

Surface energies of the study materials are presented in Table 5. Within the materials, group C specimens had higher total surface energy (*p* < 0.001) compared to SC and US in all the materials except Ti, where US surface treatment had the highest energy (*p* < 0.001). Between the materials, LDS-Press showed the highest surface energy values, followed by LDS-Glaze. The lowest surface energy values were seen in CoCr specimens.

### 3.4. Bacterial Adhesion and Biofilm Formation

**Figure 9:**
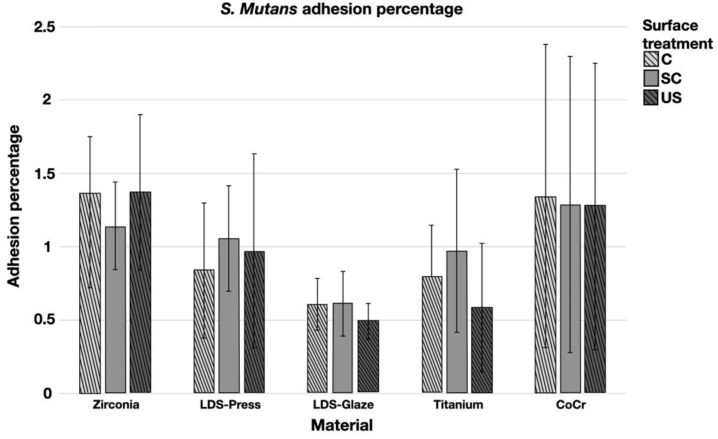
Adhesion percentage of *S. mutans* on study specimens, with different surface treatments: control (c), after scaling with a curette (SC) and after ultrasonic scaling (US).

**Figure 10:**
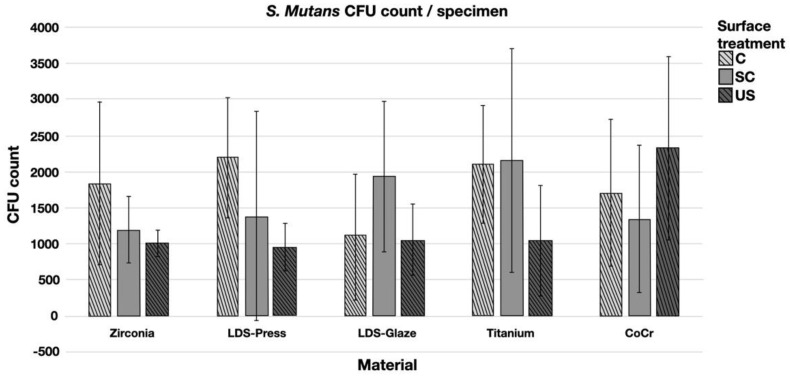
Colony forming unit (CFU) count of *S. mutans*/specimen in different surface treatment groups: control (c), after scaling with a curette (SC) and after ultrasonic scaling (US).

The results from the *S. mutans* adhesion test are presented in Figure 9. The surface treatment did not affect bacterial adhesion. However, there were some differences in bacterial adhesion between the materials. For C specimens, bacterial adhesion was significantly greater in the Zr group compared to LDS-Glaze (*p* = 0.03). For the SC specimens, there was no significant difference in bacterial adhesion between the materials (*p* = 0.084). For US specimens, Zr presented a higher adhesion percentage compared to LDS-Glaze (*p* = 0.03). There were no significant differences between other materials. Results from the biofilm accumulation test are presented in Figure 10. Neither the surface treatment within materials nor the material itself affected the biofilm accumulation.

The only significant correlations were detected between surface roughness and adhesion percentage. In the CoCr C subgroup, there was a relatively strong negative correlation (−0.715, −0.822) between the Sa and Sq values, respectively (*p* = 0.046, 0.012). However, in the SC subgroup, the correlation was positive (0.857, *p* = 0.014). Despite the surface roughness in Ti specimens being greater than in any other material, no correlations were found.

Correlation between bacterial adhesion and contact angles was seen when the contact angle measurements were done with diiodomethane. Positive correlation (0.819, *p* = 0.015) was seen in the LDS-Press C group and negative correlation was seen in the Titanium SC group (−0.833, *p* = 0.01) and the CrCo US group (−0.738, *p* = 0.037).

## 4. Discussion

The present study was conducted in order to evaluate the surface roughness of commonly used metallic and ceramic fixed prosthodontic materials after polishing or roughening with a stainless steel curette or ultrasonic scaler. The second aim was to evaluate the bacterial adhesion and biofilm accumulation of *Streptococcus mutans* on these materials with different surface treatments. The results showed that there were differences in the surface roughness values between the different materials and between the surface treatment subgroups within each material. Hence, the first null hypothesis could be rejected. However, surface roughness did not affect bacterial adhesion or biofilm formation. Bacterial adhesion was greater in Zr C and US specimens compared to LDS-Glace material with the same surface treatments. The second null hypothesis could therefore be partially rejected. The surface treatment subgroup within the materials or the material itself did not affect the biofilm accumulation.

The differences in the surface roughness values between different materials might be explained by differences in stiffness and hardness of the materials [25,26,27]. Ti and CoCr showed the highest surface roughness values and also deeper scratches on the surface after the curette and ultrasonic scaling. A similar trend has been seen in a laboratory study comparing the surface roughness of different restorative materials. Scaling the surface of type III gold alloy with a stainless steel curette induces higher surface roughness compared to zirconia and lithium disilicate [17].

Nano- and microscale surface roughness can provide more surface area for bacterial cell attachment and could therefore enhance bacterial adhesion [10]. On the other hand, there is evidence that the surface roughness (Ra) values below 0.2 µm do not affect bacterial adhesion [28,29]. This is supported by findings of the present study as almost all the roughness values were below the threshold value of 0.2 µm and the surface roughness did not affect the bacterial accumulation or biofilm formation. Highly polished surfaces were chosen, as this corresponds to the clinical situation. Only Ti SC specimens’ mean surface roughness exceeded 0.2 µm, however, no correlation with bacterial adhesion nor biofilm formation was seen. Positive correlation was detected between surface roughness and bacterial adhesion in CoCr SC specimens. However, there was no significant difference in bacterial adhesion between the SC subgroups of different materials. This might be partially explained by greater standard deviations.

Hydrophobicity of the material as well as high material surface energy can influence bacterial adhesion [12,30]. A previous study showed that lithium disilicate material had a lower bacterial adhesion percentage compared to partially stabilized zirconia when tested without saliva [31]. This finding could be replicated in the present study in the presence of saliva, when the lithium disilicate specimens were glazed (LDS-Glaze). Highly polished lithium disilicate specimens (LDS-Press) presented a similar adhesion percentage to the rest of the study materials. This is an interesting finding, because LDS-Press and LDS-Glaze had the highest surface energy values, which might attract more micro-organisms. Before surface free energy measurements, the specimens were cleaned using an ultrasonic bath with distilled water, whereas the adhesion tests were completed in the presence of saliva. Zr, CoCr and Ti specimens had higher contact angles and were considered to be more hydrophobic than lithium disilicate specimens. Differences in bacterial adhesion were only seen between Zr and LDS-Glaze materials within C and US subgroups, Zr showing a higher adhesion percentage. In addition, a previous in vitro study showed a lower adhesion percentage with other Streptococcus species on glass ceramic material compared to zirconia tested with saliva [32]. The results could be explained by the presence of saliva, as it is known that human saliva pellicles can transform the restoration surface more hydrophilic and reduce the chemical charge of the surfaces [33,34,35].

There is evidence that bacterial adhesion and biofilm formation are based on physico-chemical interactions between microbial cells and the materials’ surfaces [12]. Therefore, in the present study, the elemental composition of the surfaces was analyzed with EDX. Regardless of the fact that unexpected elements were found in the EDX analysis and roughness and wettability of the surface did not show any significant differences between Zr and LDS-Glaze materials, the bacterial adhesion was higher in Zr C and US specimens compared to LDS-Glaze. The grain size of the used zirconia material is not known. It is possible that the grain size affected the accumulation of charge in the grain boundaries. This might be associated with higher surface charge and therefore higher biofilm accumulation, which needs to be further investigated.

When testing bacterial adhesion without saliva, surface roughness seems to play a bigger role in the adhesion percentage [36]. However, this does not correspond to the clinical situation where saliva is present. According to the results of the present study, it seems that the characteristics of the material itself have more impact on bacterial adhesion, while surface roughness has only a minor role. This has been shown in previous studies for ceramic [37] as well as for resin-based materials [12]. Bacterial adhesion seems to be more dependent on the salivary pellicle and the species of the bacteria than on the material properties, and when the initial colonizers have adhered to the surface, neither surface treatment, surface material nor its properties affect the biofilm formation [37].

This in vitro study has some limitations. The sample size was chosen according to previous studies [17,38,39], however, some other studies suggest larger sample sizes [12,16]. It is known that Streptococci species are early colonizers in supragingival plaque [7,8,9]. Therefore, in order to simplify the study set-up and minimize the study variables, *Streptococcus Mutans* was the only tested bacterial species. In the clinical situation, there are certainly several bacterial species involved at the same time, which could affect the magnitude of bacterial adhesion and biofilm formation.

## 5. Conclusions

Based on this study, it can be concluded that curette and ultrasonic scaling increased the surface roughness values of LDS-Glaze, Ti and CoCr materials. Surface roughness did not affect the bacterial adhesion percentage of *Streptococcus mutans*. Zr C and US had a higher adhesion percentage compared to those of LDS-Glaze C and US. There were no differences between Zr, LDS-Glaze, LDS-Press, Ti and CoCr materials in terms of biofilm accumulation when tested with human saliva.

## Figures and Tables

**Table 1:** Study materials and manufacturing methods.

Material	Zirconia	Pressed Lithium Disilicate	Milled Lithium Disilicate, Glazed	Titanium Grade V	Cobalt Chromium
Group	Zr	LDS-Press	LDS-Glaze	Ti	CoCr
**Details**	Kyocera StarCeram Z-Al-Med (Selb, Germany)	e.max Press (Ivoclar Vivadent, Schaan, Lichtestein)	e.max CAD (Ivoclar Vivadent, Schaan, Lichtestein)	Ti6Al4V ELI–ASTM F136 (PSM Medical solutions, Gunningen, Germany)	Zenotec NP (Wieland Dental + Technik GmbH & Co. KG, Pforzheim, Germany)
**Manufacturing method**	Milled (Röders RXD5, Röders, Germany) Sintered (AmannGirrbach Ceramill Therm, Carbolite, England)	Pressed (EP5010, Ivoclar Vivadent, Schaan, Lichtenstein)	Milled (Struers Secotom-50, Copenhagen, Denmark) Glazing IPS e.max (CAD Crystall Glaze spray, Ivoclar Vivadent) Crystallized (Programat 300, Ivoclar Vivadent Schaan, Lichtestein)	Milled (DMG Ultrasonic 20, DMG Mori, Geretsried, Germany)	Milled (DMG Ultrasonic 20, DMG Mori, Geretsried, Germany)

**Table 2:** Polishing scheme of the study specimens.

Material	Zirconia	Pressed Lithium Disilicate	Milled Lithium Disilicate, Glazed	Titanium Grade V	Cobalt Chromium
Group	Zr	LDS-Press	LDS-Glaze	Ti	CoCr
**Polishing before sintering/crystallization/glazing**	Polished dry with P800 and 1200 grit silicon carbide paper (Wurth, Wuppertal, Germany)	-	Polished dry with P800 and 1200 grit silicon carbide paper (Struers, Copenhagen, Denmark), Polished with rubber tips, diamond paste (Brinell L, Renfert GmbH, Hilzingen, Germany)	-	-
**Polishing after milling/pressing/sintering/crystallization**	Diamond paste, Renfert Polish all-in-one (Renfert, Hilzingen, Germany)	Polished dry with 180 grit silicon carbide paper (Mirka GmnH, Hesse, Germany)	-	Polished wet with P800 and 1200 grit silicon carbide paper (Wurth, Wuppertal, Germany)	Polished wet with P800 and 1200 grit silicon carbide paper (Wurth, Wuppertal, Germany)
		CeraWhite polishing disc, (NTI, Kahla, Germany)			
		EVE Diacomp ultra (EVE, Ernst Vetter GmbH, Keltern, Germany)		Titapol polishing paste (Bredent medical GmbH, Senden, Germany), with soft rubber brush (Hatho GmbH, Eschbach, Germany)	Tiger brilliant polishing paste with rubber brush (Dentaurum GmbH, Ispringen, Germany)
		Dialog Vario Polish diamond paste (Schütz Dental GmbH, Rosbach vor der Höhe, Germany)			

**Table 3:** Mean elemental composition (wt.%) of the superficial layer of the study materials detected using energy dispersive X-ray analysis (EDX).

Material	Element	wt.%
**Zr**	Zr	70.60
	O	24.17
	Y	5.23
Total		100
LDS-Press *	O	48.5
	Si	31.4
	C	8.6
	K	3.1
	W	2.6
	Ce	1.7
	P	1.4
	Al	1.2
	Zn	0.9
	Mg	0.4
	Na	0.2
Total		100
LDS-Glaze *	O	42.3
	Si	28.1
	K	10.6
	Al	5.0
	W	1.7
	Zr	1.3
	Ca	1.3
	Ce	0.5
	Mg	0.5
	Na	0.2
Total		100
**Ti**	Ti	89.80
	Al	5.74
	V	4.47
Total		100
**CoCr**	Co	63.14
	Cr	30.33
	Mo	5.80
	Si	0.74
Total		100

* Lithium was not detected due to sensitivity of the EDX device.

**Table 4:** The median contact angle measurements and interquartile ranges (IQR) with different liquids.

Group		Water	Diiodomethane
Zr	C	50.9 (7.4)	38.4 (2.2)
	SC	54.9 (3.5)	38.1 (1.8)
	US	54.9 (2.9)	37.6 (1.4)
LDS-Press	C	22.2 (2.7)	40.1 (1.6)
	SC	37.3 (6.2)	42.2 (2.8)
	US	32.4 (9.3)	42.3 (3.3)
LDS-Glaze	C	43.4 (3.7)	44.8 (2.7)
	SC	39.4 (5.7)	43.1 (3.5)
	US	38.0 (6.7)	40.7 (1.9)
Ti	C	56.8 (3.1)	36.9 (1.9)
	SC	57.2 (3.6)	35.5 (1.8)
	US	51.5 (2.3)	35.3 (1.2)
CoCr	C	56.5 (3.5)	39.7 (2.6)
	SC	60.2 (6.0)	39.2 (3.0)
	US	59.7 (3.4)	39.2 (1.5)

**Table 5:** The mean surface energy SFE (SD) and its components using an Owens-Wendt approach. Differences between the materials within a surface treatment were calculated from Total SFE values.

Group		Dispersive SFE mJ/m^2^ (SD)	*p*-Value ^a^	Polar SFE mJ/m^2^ (SD)	*p*-Value ^a^	Total SFE mJ/m^2^ (SD)	*p*-Value ^a^
**Zr**	C	40.4 (0.81)	0.362	17.25 (1.2)	<0.001	57.64 (2.01)	0.002
	SC	40.58 (0.64)		14.94 (0.57)		55.52 (1.21)	
	US	40.81 (0.51)		14.84 (0.47)		55.65 (0.98)	
LDS-Press	C	39.57 (0.58)	0.014	32.53 (0.67)	<0.001	72.1 (1.25)	<0.001
	SC	38.52 (0.99)		26.01 (1.31)		64.52 (2.3)	
	US	38.43 (1.16)		28.58 (1.98)		67.01 (3.14)	
LDS-Glaze	C	37.1 (0.92)	0.001	23.22 (0.83)	0.002	60.32 (1.75)	<0.001
	SC	38.0 (1.23)		25.05 (1.26)		63.32 (2.49)	
	US	39.25 (0.69)		24.8 (1.32)		64.06 (2.01)	
Ti	C	41.12 (0.69)	0.013	13.69 (0.51)	<0.001	54.81 (1.2)	<0.001
	SC	41.79 (0.67)		13.0 (0.53)		54.79 (1.2)	
	US	41.89 (0.46)		16.35 (0.41)		58.24 (0.87)	
CoCr	C	39.77 (0.94)	0.738	14.36 (0.62)	<0.001	54.13 (1.57)	0.011
	SC	40.0 (1.1)		12.2 (0.88)		52.2 (1.99)	
	US	40.05 (0.55)		12.46 (0.51)		52.51 (1.05)	
*p*-value ^b^	C					<0.001	
	SC					<0.001	
	US					<0.001	

^a^ differences between surface treatments within material; ^b^ differences between materials within surface treatment.

## Data Availability

The data presented in this study are available on request from the corresponding author.
